# Continuous Performance Tasks: Not Just About Sustaining Attention

**DOI:** 10.1044/2015_JSLHR-L-15-0068

**Published:** 2016-06-01

**Authors:** Hettie Roebuck, Claudia Freigang, Johanna G. Barry

**Affiliations:** a MRC Institute of Hearing Research, Nottingham Clinical Section,Queen’s Medical Centre, United Kingdom; b Waisman Center, University of Wisconsin, Madison

## Abstract

**Purpose:**

Continuous performance tasks (CPTs) are used to measure individual differences in sustained attention. Many different stimuli have been used as response targets without consideration of their impact on task performance. Here, we compared CPT performance in typically developing adults and children to assess the role of stimulus processing on error rates and reaction times.

**Method:**

Participants completed a CPT that was based on response to infrequent targets, while monitoring and withholding responses to regular nontargets. Performance on 3 stimulus conditions was compared: visual letters (X and O), their auditory analogs, and auditory pure tones.

**Results:**

Adults showed no difference in error propensity across the 3 conditions but had slower reaction times for auditory stimuli. Children had slower overall reaction times. They responded most quickly to the visual target and most slowly to the tone target. They also made more errors in the tone condition than in either the visual or the auditory spoken CPT conditions.

**Conclusions:**

The results suggest error propensity and reaction time variations on CPTs cannot solely be interpreted as evidence of inattention. They also reflect stimulus-specific influences that must be considered when testing hypotheses about modality-specific deficits in sustained attention in populations with different developmental disorders.

Attention is a complex construct that has been implicated in disorders such as attention-deficit/hyperactivity disorder (ADHD). Of the many tasks developed to assess different aspects of attention, the continuous performance task (CPT) is cited as the most frequently used in both clinical and research contexts (Riccio, Reynolds, Lowe, & Moore, 2002). The task is conceptualized as a measure of both selective and sustained attention, though to a lesser extent it may also gauge aspects of impulsivity. It is based on a simple go–no go design, but many variants of it have been described (see Corkum & Siegel, 1993; Ebert & Kohnert, 2011; Riccio et al., 2002). As a consequence, it is difficult to generalize findings across studies, and there is a real need for consistent, comparative study of common populations (Riccio et al., 2002). This article describes such a study involving typically developing children and adults. The primary aim was to assess the impact of stimulus choice on observations of CPTs.

In the following, we describe the CPT paradigm and briefly review what is currently known about factors affecting performance. Most research with CPTs focuses on ADHD, however modality-specific deficits in sustained attention have also been implicated in other developmental disorders such as specific language impairment (SLI) and auditory processing disorder (APD). As we show, the validity of this claim is difficult to assess due to the wide variety of stimuli used across studies. We conclude this section by summarizing different theories posited to explain lapses of attention. These highlight conflicting views regarding whether stimulus processing plays a part in this phenomenon. They contribute to the development of the key hypotheses for our research.

## The CPT and the Factors That Influence It

The CPT first came to prominence when Rosvold, Mirsky, Sarason, Bransome, and Beck (1956) demonstrated its sensitivity to brain damage, and the task has since been used to quantify ability to sustain attention over time in a range of clinical populations. Essentially, the CPT can be described as a continuously changing stream of stimuli in which a rarely occurring target stimulus is embedded. The participant is required to either only respond to this target (the standard CPT paradigm) or only withhold responses to it (the sustained attention to response task CPT variant). Performance is operationalized in terms of error rates (lapses in attention) and reaction times (processing time).

Apart from the specific task requirements (i.e., withhold response or respond to a specified target), CPTs can vary along one or more of a number of other dimensions, including stimulus type and modality, the duration of the task, the relative proportion of targets to nontargets, and the rate of stimulus presentation.

There have been a number of studies on the factors that might influence performance on the CPT (Ballard, 1996; Corkum & Siegel, 1993; Riccio et al., 2002). From all these studies, it is apparent that performance is sensitive both to factors external to the CPT (i.e., time of day, personality, gender, presence or absence of noise, the offering of rewards) as well as to factors internal to it (i.e., the specific combination of task parameters used). The wide variation in test conditions and test designs, together with the susceptibility of overall performance to these variables, makes it difficult to explain inconsistencies in findings between studies. Moreover, at least in the context of ADHD, a task’s internal factors seem to interact with one another to determine the degree to which individual CPTs are sensitive to the presence of attention deficits (Corkum & Siegel, 1993).

## The Use of CPTs to Study Other Developmental Disorders

Corkum and Siegel’s (1993) review focused on tests involving visual stimuli. This is reasonable in the context of ADHD, where attention deficits define the disorder and may be observed regardless of modality (American Psychiatric Association, 2013). However, more recently, deficits in sustained attention have also been noted in SLI (reviewed in Ebert & Kohnert, 2011) and APD (Moore, Ferguson, Edmondson-Jones, Ratib, & Riley, 2010). It has been suggested that these deficits are specific to the auditory domain (APD) or may even be restricted to a type of stimulus within a modality (e.g., Ebert & Kohnert, 2011, with respect to SLI).

Interest in the possibility of deficits in sustained attention in APD is relatively recent (although see Barry, Tomlin, Moore, & Dillon, 2015; Gyldenkærne, Dillon, Sharma, & Purdy, 2014; Tomlin, Dillon, Sharma, & Rance, 2015). However, in the case of SLI, a number of studies have been published that tested for the presence of these deficits in the disorder. These studies have used a wide range of stimuli presented in either the auditory modality (e.g., Bishop & Norbury, 2005 [tones]; McArthur & Bishop, 2004 [noises]) or the visual modality (e.g., Finneran, Francis, & Leonard, 2009 [shapes]; Spaulding, Plante, & Vance, 2008 [animations]). Studies have also used stimuli combining modality and linguistic influences; examples include auditory-linguistic stimuli such as syllables (Greyerbiehl, 1981) or words (Das & Äystö, 1994).

The conclusions from these studies are conflicting (Ebert & Kohnert, 2011). Two studies testing for deficits across modalities reported auditory-specific deficits (Noterdaeme, Amorosa, Mildenberger, Sitter, & Minow, 2001; Spaulding et al., 2008), whereas other studies reported no evidence of such deficits (Hanson & Montgomery, 2002), and still others provided evidence suggesting the difficulties extended to the visual modality (Finneran et al., 2009). With such variability in the stimuli chosen to represent a modality, it is difficult to assess the role of these choices in explaining some of the inconsistencies in findings across CPT studies.

## Theories to Explain Lapses of Attention

Researchers may have well-motivated reasons for the specific design of the stimuli that they use in their studies, but, to our knowledge, there is no explicit interest in the impact of these choices on cross-study consistency in findings. The apparent lack of interest in stimulus choice may reflect the fact that ability to sustain attention is seen as being relatively separate from stimulus processing and primarily associated with fluctuations in resource capacity (Kahneman, 1973). Although evidence has recently been found for effects on response rates across modalities (Seli, Cheyne, Barton, & Smilek, 2012), theories specifically aimed at explaining failures in sustained attention (i.e., lapses in attention) differ in whether stimulus processing has contributed to the phenomenon.

There are two main families of theories for explaining failures in sustained attention. Proponents of underload theories (Nachreiner & Hanecke, 1992) argue that they reflect a decrease in arousal induced by low task demand. These theories predict that CPT performance would be relatively immune to stimulus-specific effects. They contrast with overload theories. In these theories, the process of sustaining attention is seen as being cognitively demanding due to the inherent workload associated with continuous processing of stimuli and subsequent response selection (Hitchcock, Dember, Warm, Moroney, & See, 1999). Lapses in attention are thought to reflect a periodic expenditure of available resources (Head & Helton, 2014). Because different stimuli may be inherently more or less cognitively demanding to process, these models predict that CPT error rates and reaction times would associate to some extent with stimulus-specific variations in processing cost.

In the context of developmental disorders, where stimulus modality effects are at issue, underload and overload theories would have different implications for our understanding of why deficits in sustained attention are observed. However, to truly address questions about underlying causes for lapses of attention in different developmental disorders, CPTs must be equated for stimulus-processing difficulty both within and between modalities. This requires comparison of performance on matched CPTs in a group where confounds of attention or processing difficulties are excluded. It is then possible to tease out contributions to performance from stimulus-processing demands.

## The Study Design and Hypotheses

In this study, to address the question of whether or how stimulus type affects CPT performance, we compared observations of adults and children with no history of difficulties with listening, language, or attention both between modalities (auditory vs. visual) as well as within a modality (auditory–nonspeech vs. auditory–linguistic, i.e., speech). The children were aged from 8–12 years. This is a typical age range for many developmental studies (Corkum & Siegel, 1993) and covers a noted turning point in development on CPT performance (Parasuraman & Davies, 1984). The inclusion of the adults provided a measure of end point ability. We predicted that adults would be both faster and more accurate than children on all tasks (Carriere, Cheyne, Solman, & Smilek, 2010).

Between modality, we compared performance on visual versus auditory instantiations of X and O. Within the auditory modality, we compared performance between the spoken X and O stimuli, with nonlinguistic pure tones. Performance on the three CPT conditions was operationalized as (a) numbers of errors and (b) reaction time. The first measure indicates possible failures in attention, whereas the second is complex to interpret. It reflects speed of orientation to stimuli as well as effects due to stimulus-processing demands.

No differences in performance between stimulus conditions for either the adults or the children would support underload theories of attentional lapses (Nachreiner & Hanecke, 1992). By contrast, differences in performance either within or between modalities would suggest that performance on CPTs is sensitive to stimulus-specific processing demands. This would offer evidence in support of overload theories regarding failures in attention maintenance (Head & Helton, 2014).

## Method

### Ethics Statement

This study was approved by the Nottingham Research Ethics Committee 1. Informed written consent was received from all participants who took part in the study. All experimental procedures complied with the British Psychological Society Code of Ethics and Conduct and with the World Medical Association Helsinki Declaration as revised in October 2008.

## Participants

All participants were native speakers of English. To be included in the study, children had to meet definitional criteria for typical development—that is, normal hearing (bilateral pure-tone hearing thresholds of 25 dB HL or better for frequencies 250, 500, 1000, 2000, and 4000 Hz), nonverbal IQ (NVIQ) ≥ 80 (Wechsler Abbreviated Scale of Intelligence [WASI]; Wechsler, 1999), no history of difficulties with attention (Conners’ Rating Scales–Revised score ≤ 60; Conners, 2001), overall language abilities in the normal range (Children’s Communication Checklist, Version 2, General Communication Composite score ≥ 55; Bishop, 2003), normal listening abilities (Evaluation of Children’s Listening and Processing Skills scaled score ≥ 6 for factors capturing listening and language; Barry & Moore, 2014), and no other reported difficulties (family history questionnaire developed in-house). Adults had to have a normal NVIQ (same WASI minimum as the children), normal attention (Adult Self-Report Scale–V1.1 Screener; Adler, Kessler, & Spencer, 2003), and no reported history of difficulty with language or listening (family history questionnaire).

Twenty-two adult participants aged between 18 and 50 years and 31 children aged between 8 and 12 years were initially recruited to the study. Eleven children were subsequently excluded because they did not meet criteria for participation (eight due to clinically significant difficulty on at least one of the screening questionnaires, two due to pure-tone average hearing thresholds > 25 dB HL, and one due to an NVIQ < 80). Two adults were excluded. One did not complete the entire test battery and the other had hearing thresholds > 25 dB HL. Details for all remaining participants are summarized in Table 1.

## Procedure

The participants undertook a large test battery designed to assess a range of cognitive skills. The full test battery took 2.5 hr to complete. Testing was therefore broken up into two sessions each lasting approximately 1 hr and 15 min, with a minimum break of .5 hr between sessions. Additional smaller breaks were permitted as needed. For the purposes of this research, we only report the results from the CPT, WASI, and digit span tasks.

### Assessment of Ability to Sustain Attention

Sustained attention was assessed using the test parameters from the Tests of Variables of Attention (TOVA). This CPT is frequently used to support the diagnosis of ADHD (Leark, Dupuy, Greenberg, Kindschi, & Hughes, 2011). The parameters chosen for the task were designed to optimize the detection of inattentiveness by challenging the participant to sustain attention to infrequent targets while monitoring and withholding responses to regularly presented nontargets.

In this study, as per procedures outlined by Leark et al. (2011) for testing inattention, 22.5% (*n* = 72) of the stimuli were targets and 77.5% (*n* = 252) were nontargets. The order of their presentation was randomized. Participants were instructed to respond by pressing a button on a button box as soon as they saw or heard the target stimulus. They were instructed to withhold a response (i.e., do nothing) when they saw or heard the nontarget. The experiment was presented through PsychoPy version 1.78 (Peirce, 2007).

Three different stimulus conditions were included: two visual shapes/letters (X, O), two spoken analogs (“Ex” and “Oh”), and two pure tones (“low” C, 261.6 Hz; “high” G, 392 Hz; Leark et al., 2011). Spoken sounds were recorded for a male speaker of standard British English in a soundproof booth, and sound levels were normalized using the software Audacity (Audacity Team, http://audacity.sourceforge.net/). The stimuli used in the tone task were also generated in Audacity. In the visual condition, the letters X and O were presented centrally on a black screen in white Arial font with a 110-point font size.

The order of presentation of the three stimulus conditions was counterbalanced. To exclude the possibility of consecutive presentation, they were interleaved with tests forming part of the larger testing battery.

Each CPT condition lasted just over 11 min. All stimuli, regardless of condition, were presented for 200 ms with an interstimulus interval (ISI) of 2,000 ms. The 200-ms stimulus duration ensured that the complex speech sounds were recognizable while still being within the optimal range for sensitivity to attention difficulties (Corkum & Siegel, 1993). A long ISI of 2,000 ms supports increased response accuracy while minimizing false alarms (Sykes, Douglas, Weiss, & Minde, 1971). Both auditory conditions were presented at 65 dB SPL.

During testing, participants were seated in a quiet room and completed the experiment on a 15-in. laptop with a screen resolution of 1,024 × 768 pixels. A uniform black screen was presented during the experiment. A practice session was provided prior to the presentation of each stimulus condition. This was to ensure that participants understood the task and were familiar with both the target (X or “Ex” or high) and the nontarget (O, “Oh” or low) for each stimulus condition. The practice session consisted of randomized presentations of an equal ratio of targets and nontargets, presented at the same ISIs and durations as the stimuli in the experimental conditions. Feedback, in the form of a happy or sad face, was given in the practice session to ensure the participant understood when they were doing the task correctly. If participants scored fewer than seven out of 10 correct responses in the practice session, the instructions were explained again and the practice session repeated. Five children needed to repeat the practice session for the tone CPT condition only. The experiment was initiated once the participant demonstrated that he or she had understood and could perform the task reliably. No feedback was given during testing.

### Cognitive Testing

To test for variations in cognitive contribution to the different CPT variants, performance on measures of short-term memory and NVIQ were collected. Digit span was used to measure serial short-term and working memory capacity. Participants were presented with a series of digits (one per second), and they were requested to repeat the series immediately after presentation. Two trials of each series length were presented before increasing the length by one. The test was halted if participants were unable to recall two digit sequences of the same length. The sequences of digits ranged in length from two to nine digits. The process was repeated for backward digit span (sequence lengths ranged from two to eight digits) where participants were required to recall the series of digits in reverse order. Participants were awarded 1 point per correctly repeated digit sequence.

NVIQ was assessed using both performance subtests from the WASI. These were (a) matrix reasoning, where a picture is selected to best fit in the gap of a series of images; and (b) block design, where specific patterns are created using red and white blocks. The scores from the two subtests were summed and converted to a standard score.

### Statistical Analyses

Performance (ability to sustain attention) was assessed using measures of response accuracy and reaction time. Correct responses to the targets were averaged to calculate reaction times. Responses < 100 ms were considered anticipatory and were excluded. The onset of the next target (2,000 ms) provided the upper cutoff limit for responding. Error propensity was assessed with respect to two error types: omission errors (missed targets) and false alarms (responses to nontargets). Error propensity for both error types was at ceiling in the adult group, and only reaction times to correct detection were considered further for this group.

All analyses were performed within SPSS Version 21 for Windows (2012). Omission errors, false alarms, and reaction times were all nonnormally distributed (Kolmogorov– Smirnov test, all *p* < .001) and had a positive skew (see Table 2). They could not be transformed to achieve a normal distribution, and differences among conditions were tested using the nonparametric Friedman’s analysis of variance (ANOVA). For the correlation analyses, we report parametric Pearson’s *r* rather than the nonparametric Spearman’s rho, given that there was no reason to expect a nonlinear monotonic relationship between our variables. There were also no obvious outliers, and a nonparametric permutation test of the null hypothesis that the two variables are uncorrelated indicated a nonvanishing Pearson correlation (*p* = .029). This suggested the test would be robust against the distribution of our data.

## Results

### Role of Modality and Stimulus Differences on the Ability to Sustain Attention

**Figure 1:**
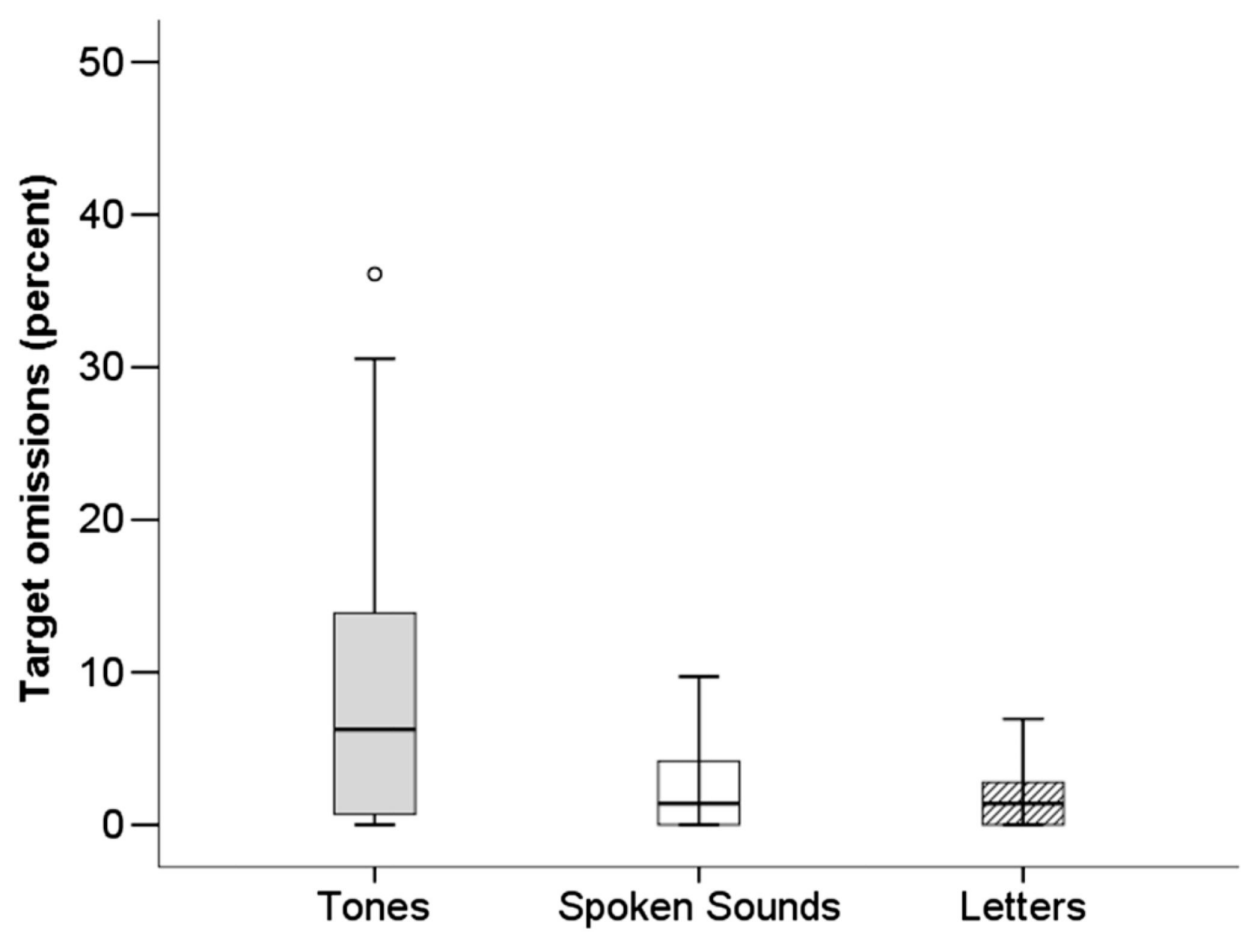
Box plots summarize omission errors (i.e., targets missed) for each stimulus condition in the child group. The black bars indicate median scores, the top to bottom edges of the boxes incorporate the interquartile range of performance, and the whiskers show the range of scores. Outliers are identified as falling outside 1.5 (°) times the box length from the upper and lower edges of the boxes.

In this first analysis (Figure 1), a repeated measures Friedman’s ANOVA was performed to assess effects of stimulus type on omission errors in the children (1 × 3 conditions: visual letters, spoken sounds, or nonspeech tones; see Table 2). There was a significant effect of condition, χ^2^(2) = 8.52, *n* = 20, *p* = .014. Post hoc tests with Bonferroni adjustments for multiple comparisons (*p* is significant at .05 / 3 = .017) indicated significantly more errors in the tone compared with the visual letters (*p* = .002) and spoken sound conditions (*p* = .006). There was no difference between these latter two conditions (*p* = .252).

### Role of Modality and Stimulus Differences on Ability to Withhold Responses to Nontargets

**Figure 2:**
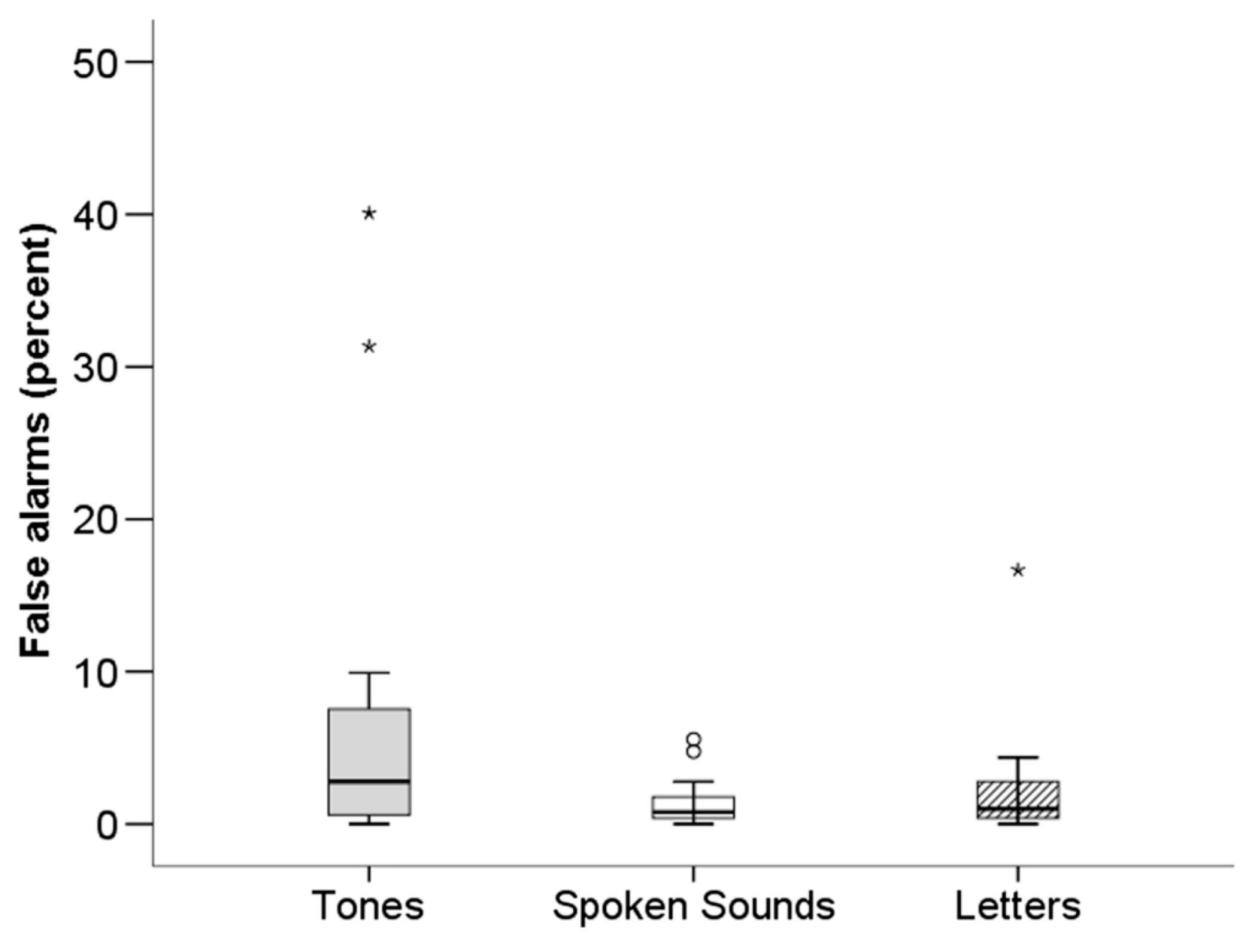
Box plots summarize false alarms for each stimulus condition in the child group. The black bars indicate median scores, the top to bottom edges of the boxes incorporate the interquartile range of performance, and the whiskers show the range of scores. Outliers are identified as falling outside 1.5 (°) and 3 (*) times the box length from the upper and lower edges of the boxes.

Errors withholding responses (false alarms) reflect tendencies toward impulsivity. A Friedman’s ANOVA compared false alarms in the children across condition (1 × 3: visual letters, spoken sounds, nonspeech tones). Results for the children are summarized in Figure 2 and Table 2. Significant differences across stimulus conditions were observed, χ^2^(2) = 9.62, *n* = 20, *p* =.008. Post hoc analyses with Bonferroni correction for multiple comparisons (*p* was significant at .017) indicated significantly more errors for the nonspeech tone condition compared with the spoken sound condition (*p* = .003). However, there was no difference between the visual letter or spoken sound conditions (*p* = .419) or between the nonspeech tones and visual letter conditions (*p* = .048).

Overall, regardless of task demand (i.e., respond to target or withhold response to nontarget), children showed increased error propensity in the nonspeech tone condition relative to other CPT stimulus conditions. In addition to task-specific demands, errors observed also reflected stimulus-specific effects.

### Role of Modality and Stimulus Differences on the Speed of Response to Targets

**Figure 3:**
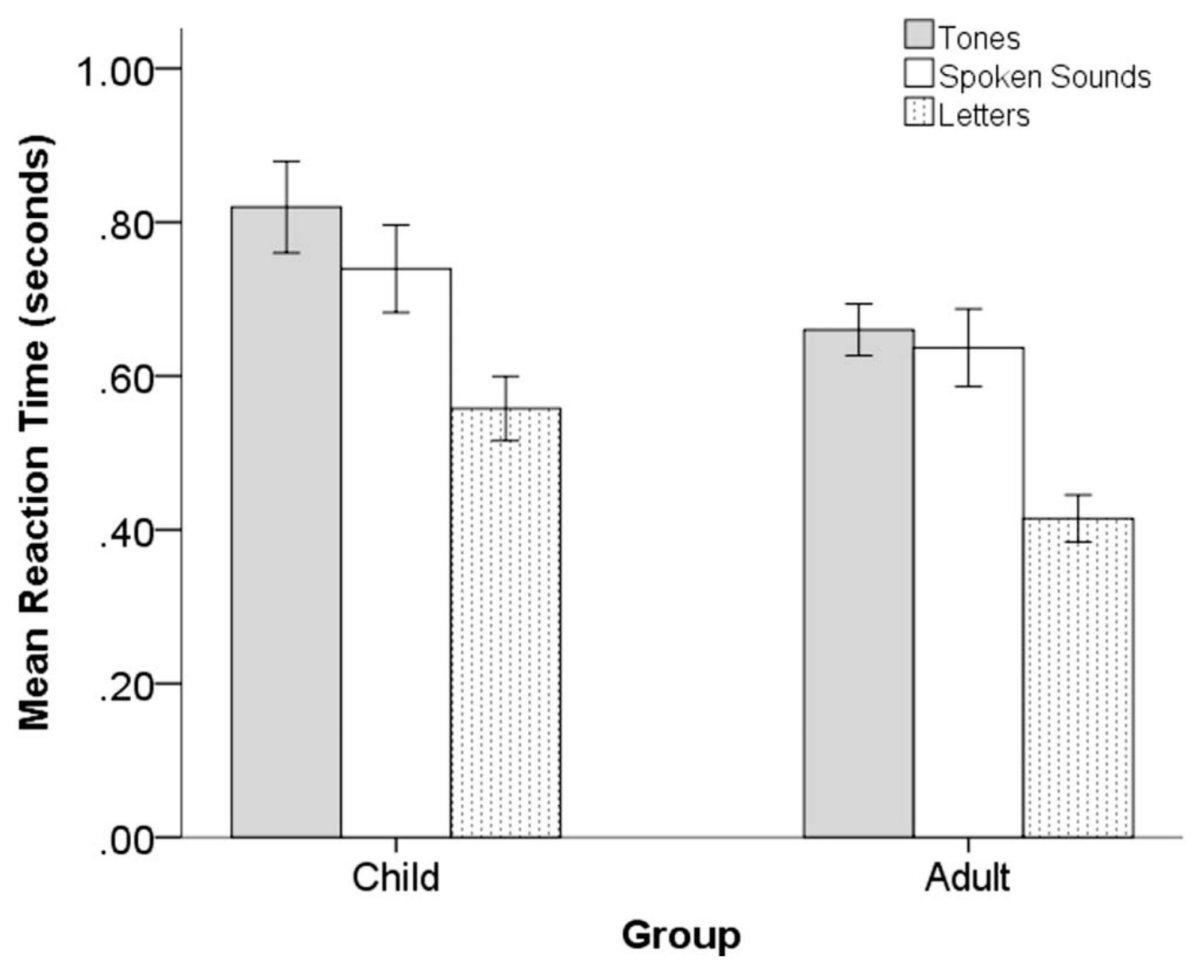
Median reaction time (seconds) to targets in the three stimulus conditions (auditory: tones, spoken sounds; visual: letters) for the two groups of participants. Error bars depict the 95% confidence intervals.

Reaction time is a complex measure that reflects both speed of orientation to stimuli as well as processing demand. Whereas adults were at ceiling for response errors on the three CPT conditions, they showed stimulus-specific differences in reaction time (Figure 3, Table 2).

A Mann–Whitney *U* test compared between-groups differences (adult vs. child) in reaction time for correctly identified targets. Overall, children had slower reaction times than adults (*U* = 305.0, *p* = .004). Within-group differences were explored using separate group-specific Friedman’s repeated measures ANOVA (Bonferroni correction for multiple comparisons was significant at *p* < .017).

Adult reaction time across the three CPT stimulus conditions indicated a significant effect for condition, χ^2^(2) = 32.4, *n* = 20, *p* < .001. The fastest reaction times were observed for the visual letter CPT condition (*p* < .001, for both speech and tone). The two auditory conditions did not differ significantly from each other (*p* = .171; see Table 2, Figure 3).

Child reaction times were assessed in the same way and also showed a significant main effect for condition χ^2^(2) = 30.1, *n* = 20, *p* < .001 (see Table 2, Figure 3). As for the adults, fastest reaction times were observed in response to visual letters (*p* < .001 for both speech and tone). However, in this group, there was also a difference between the two auditory conditions, with responses to spoken sounds being significantly faster than responses to nonspeech tones (*p* = .015).

### Role of Task Order on Performance

The order of the stimulus conditions was counter-balanced to account for the influence of practice effects. However, it is still interesting to consider the effect of task experience on performance (i.e., does prior exposure to one CPT condition affect performance on subsequent conditions?).

Omission errors were classified according to order of presentation of each stimulus condition (first, second, or third) and compared using a Kruskal–Wallis test. No significant effect was observed for order of presentation regardless of stimulus type: tones, χ^2^(2) = 2.10, *p* = .35; visual letters, χ^2^(2) = 3.22, *p* = .20; or spoken sounds, χ^2^(2) = 0.66, *p* = .72. The same was true for reaction time: tones, χ^2^(2) = 1.72, *p* = .42; visual letters, χ^2^(2) = 5.64, *p* = .06; or spoken sounds, χ^2^(2) = 3.78, *p* = .15. Overall, a task’s internal factors specific to the stimulus CPT condition, rather than task experience, modulated performance.

### Cognitive Contributions to Performance

In this study, all internal parameters of the tasks were held constant across conditions to reveal potential stimulus-specific effects. The longer reaction times and greater error propensity observed for the tone condition in the children (Figures 1 and 2) suggest a change in processing demand that must be specific to the stimulus.

To explore the nature of these demands, a series of correlations with age, serial short-term memory (digit span forward), working memory (digit span backward), and NVIQ were performed for each of the three CPT conditions. We predicted reduced error propensity with increasing age (Carriere et al., 2010), greater memory (Kail & Park, 1994; Gathercole et al., 2008), and higher NVIQ.

**Figure 4:**
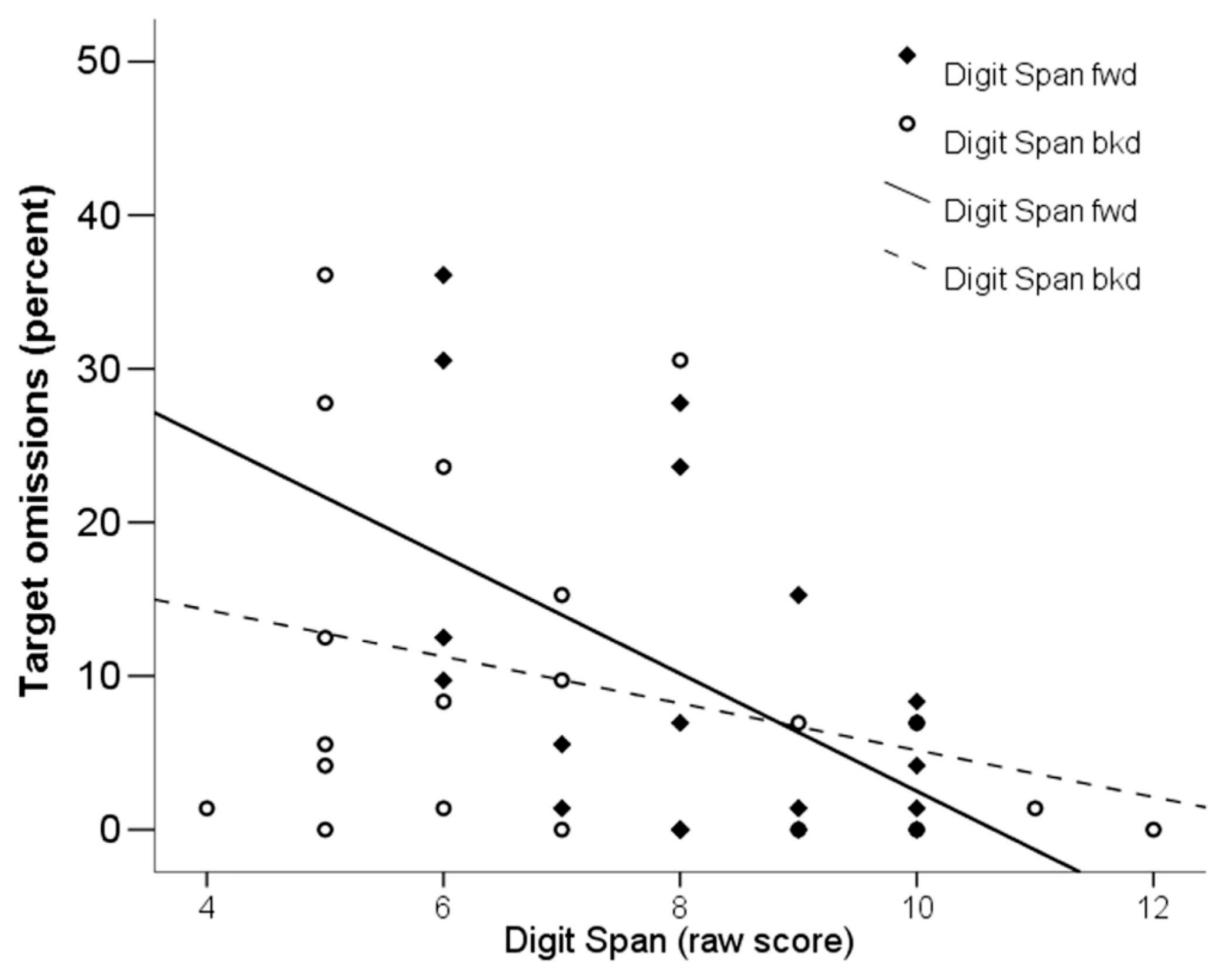
Correlations between a measure of serial short-term memory (digit span forward), working memory (digit span backward), and percentage targets missed in the tone task in the child group.

All correlations are summarized in Table 3. Figure 4 displays the distribution of the data for findings with respect to the digit span measures. Three correlations with *p* < .05 (bolded in Table 3) were observed. Due to the number of tests performed, two of these did not survive correction for multiple comparison (Bonferroni, *p* < .004). The direction of correlation, though, was consistent with prediction.

Significant inverse correlations with age were observed for both auditory CPT conditions but not for the visual one. This suggests a prolonged developmental trajectory for the processing of auditory relative to visual stimuli.

A significant correlation (uncorrected) was also observed between omission errors on the tone CPT and serial short-term memory (digits forward; Figure 4), where, as predicted, lower digit span scores were correlated with increased omission errors. The presence of a correlation for only this condition suggests it poses greater demands on serial short-term memory than the other two conditions do. However, age and digit span forward also correlated (*r* = .63, *p* = .002), and to explore the influence of short-term memory on error propensity on a CPT further, it would be necessary to power the study sufficiently to control for age effects.

There was no evidence of correlation with omission errors for any of the three CPT conditions and either digit span backward or NVIQ. This suggests performance on the tasks did not tap into higher level cognitive factors such as executive function or working memory.

## Discussion

The influence of stimulus type on reaction times and error propensity for CPTs has largely been ignored in past research. The lack of explicit interest in this aspect of the test design possibly reflects the view that fluctuations in attention are commonly thought to reflect some form of decoupling from active on-task attention (e.g., Nachreiner & Hanecke, 1992). In this study, we assessed the validity of this view by assessing the influence of stimulus type of CPT performance in typically developing adults and children while keeping all other task parameters constant. We showed how, even in these groups, the inclusion of tone stimuli in the design imposes an extra task demand that is reflected in slower reaction times as well as increased error propensity in the children, regardless of the task requirement (response [omission] vs. inhibition [false alarm]). Our findings provide support for overload (Head & Helton, 2014; Hitchcock et al., 1999) rather than underload influences on CPT performance (Nachreiner & Hanecke, 1992).

In the following, we consider the reasons why tonal stimuli introduce extra processing demands. We conclude by considering the implications of our findings for understanding more about the nature of deficits in sustained attention in developmental disorders.

When considering the design of a CPT, two factors regarding stimulus choice predominate in the developmental literature: (a) the modality of the stimuli (visual vs. auditory; e.g., Noterdaeme et al., 2001; Spaulding et al., 2008) and, less frequently, (b) the influence on performance of higher level factors like language or reading abilities (e.g., Ebert & Kohnert, 2011). Thus, for example, the TOVA—a CPT frequently used to support assessment of ADHD (Leark et al., 2011)—explicitly minimizes higher level processing effects associated with cultural differences and language/reading abilities by using pure-tone stimuli for the auditory CPT condition. These stimuli are designated *high* and *low* in the task instructions. The visual condition uses shapes that are also designated *high* versus *low* on the basis of their location on a screen. The TOVA was clearly designed with the aim of making the auditory and visual conditions as comparable as possible in terms of processing demands. Nonetheless, it is notable that norms for the visual TOVA are available for children aged 4 years and older, whereas they are available for the auditory TOVA only for children older than 5 years. In part, as our data suggest, the difference in available norms can be attributed to developmental differences in the efficiency of processing of auditory versus visual stimuli.

Correlations were observed between age and error propensity in both auditory tasks but not in the visual task. Assuming the auditory and visual X and O stimuli are fully balanced for processing demand, this suggests a prolonged developmental trajectory for information processing in the auditory modality compared with the visual modality. However, the relationship between error propensity and age was more marked for the tonal stimuli. This, together with the greater individual variation in scores on the tone variant of the CPT for both omissions and false alarms, suggests developmental differences are only part of the story. Tones, for all their apparent simplicity, must offer some form of extra processing demand. Similar concerns have been raised in the auditory processing literature, where the focus is on determining just noticeable differences in frequency discrimination thresholds (e.g., Barry, Weiss, & Sabisch, 2013; Halliday, Taylor, Edmondson-Jones, & Moore, 2008). The question is, why are tasks involving pure tones more cognitively demanding?

Both in children and adults, wide individual differences in frequency discrimination thresholds have been attributed to increased encoding demands associated with tonal stimuli (Bishop, Carlyon, Deeks, & Bishop, 1999). For this reason, frequency discrimination, particularly in children, is preferentially tested using some form of odd-ball, three-interval forced-choice paradigm. Such paradigms minimize the need to assign specific attributes to stimuli as part of the decision-making process. By contrast, the CPT used here explicitly requires participants to support decision making by associating an attribute (high vs. low) to the target and nontarget. Although the aim in using these terms may have been to help participants, it is also possible that the choice of these labels contributed to the higher error propensity observed for the tone CPT condition. The labels are not concrete identifiers; rather, they refer to a relative auditory quality holding between the target and nontarget tones that must be verified before providing (withholding) a response. This process of verification potentially has an impact on speed of decision making, leading to the relatively slow reaction times observed in this condition. It is also likely to affect error propensity, given that children are known to have greater difficulty labeling pitch using such terms compared with more concrete terms like *aigu* (sharp) and *grave* (flat; Costa-Giomi & Descombes, 1996).

Apart from increased processing demand associated with using labels on the basis of relative auditory qualities, the CPT used a long ISI of 2,000 ms. Listeners therefore had to rely to some extent on their short-term memory capacity to support task performance. Cowan’s (1984) model of information processing suggests that short-term memory is not an isolated system. Individuals draw on information stored in long-term memory to support or enhance memory traces held in short-term memory. Such a mechanism would be available for the processing of auditory/visual X and O stimuli but would not be available to support the processing of tones (i.e., listeners are almost wholly reliant on their short-term memory capacity when deciding whether they had heard a target or nontarget).

Short-term memory capacity increases (Kail & Park, 1994) and error propensity on CPTs decreases (Carriere et al., 2010) as children mature. This complicates the interpretation of any correlation analysis. Nonetheless, an association was observed between increased error propensity and poorer serial short-term memory, particularly in the younger children in the study. No similar association was apparent with the spoken variant of the CPT.

The CPT is designed to capture tendencies toward inattention by minimizing all other cognitive demands. One would not therefore expect any associations with higher cognitive abilities that are based on task requirements, though symptoms of inattention have been noted in children with poor working memory (Gathercole et al., 2008). This might be expected to emerge in individual variations in error propensity in the CPTs. In fact, no such associations were observed, nor were there any associations with NVIQ. Thus, at least in typically developing children, beyond ability to maintain attention, the primary factors influencing observations on the CPT paradigm used in this study were stimulus specific. Moreover, as revealed in the tone condition, these factors reflected a complex interaction between memory-trace maintenance in short-term memory, a relatively low-level factor, and efficient stimulus labeling, a higher level factor.

The discussion to now has been about understanding the impact of stimulus choice on error propensity and reaction time. However, more broadly, conflicting evidence has been presented regarding the modality specificity of difficulties maintaining attention in developmental disorders. Our data suggest stimulus choice may be a factor in these mixed findings.

A review of Ebert and Kohnert’s (2011) meta-analysis of these studies indicates wide variation even within a modality. For example, in the auditory modality, tones (Bishop & Norbury, 2005), syllables (Greyerbiehl, 1981), words (Das & Äystö, 1994), and environmental noises (McArthur & Bishop, 2004) have been used. The meta-analysis suggested an effect was more likely to be seen with auditory-linguistic stimuli, but it was difficult to disentangle basic cognitive functions from language demands. An important conclusion from our own study with typically developing children is that stimulus choice is not a trivial issue. Some stimulus types (herein, tones) introduce higher processing costs, which have an impact on error propensity and reaction time. It is thus important to consider possible differences in processing demand when testing questions regarding within- and between-modalities effects on sustained attention. Both physical differences (e.g., loudness, frequency difference, brightness, size) and cognitive differences between stimuli (e.g., memory load, ease of labeling) should be evaluated. Cross-condition comparison in groups without underlying difficulties (as described herein) should be performed to ensure that any effects observed are specific to the demands of the task. Finally, developmental groups may have a complex range of underlying difficulties, which also may influence findings from CPTs. It is thus important when probing specifically for sustained attention deficits to ensure the appropriate cross-condition comparisons are included to ensure that the hypotheses of interest are reliably assessed.

Finally, we believe our results have relevance for broadening understanding of processes captured by CPTs. These tasks are designed to capture an individual’s propensity for disengaging from a task. However, stimulus choice clearly affects observation. Although we have not strictly varied resource requirements, we would argue that the nature of the stimulus is a relevant component contributing to on-task demand for cognitive resources. We suggest that failures in performance on CPTs are not only a reflection of task decoupling (an inherently supramodal effect) but also affected by resource demands (Head & Helton, 2014), including those associated with stimulus processing.

## Conclusion

CPTs are deceptively simple tasks that have been designed to capture difficulties in maintaining attention. This study shows how error propensity and reaction time on a CPT do not solely reflect fluctuations in attention/alertness but also suggest that individual variations in processing resources may be relevant (Head & Helton, 2014). The effects of these more complex contributions should be minimized by choosing stimuli with low processing demands. When addressing questions to do with characteristics specific to the stimulus (e.g., modality or linguistic effects), the comparability of processing demands between conditions should also be considered.

## Figures and Tables

**Table 1:** Summary of participant details according to gender (female [F], male [M]), and the mean (*M*) and standard deviation (*SD*) for age (years) and nonverbal IQ (NVIQ) standard score (SS).

	Gender	Age (years)	NVIQ (SS)

Group	F:M	*M* (*SD*)	*M* (*SD*)
Adult	9:11	26 (10)	111 (12)
Child	8:12	9.8 (1.1)	110 (12)

**Table 2:** Summary of descriptive statistics: Mean (*M*) and standard deviation (*SD*), median and interquartile range (IQR), and skewness for omission errors, false alarms, and reaction times for each condition and both age groups.

Group	Condition	M (*SD*)	Median	(IQR)	Skewness
Percentage of omission errors					
Children	Tone	9.6 (11.3)	6.3	(13.2)	1.14
	Spoken	2.8 (3.1)	1.4	(4.2)	1.14
	Letter	2.0 (2.3)	1.4	(2.8)	1.09
Adults	Tone	0.5 (1.0)	0.0	(0.0)	1.71
	Spoken	0.7 (1.8)	0.0	(0.0)	2.72
	Letter	2.6 (5.6)	0.0	(2.1)	2.48
Percentage of false alarms					
Children	Tone	6.5 (10.6)	2.8	(7.0)	2.33
	Spoken	1.4 (1.5)	0.8	(1.4)	1.53
	Letter	2.1 (3.7)	1.0	(2.4)	3.32
Adults	Tone	0.3 (0.5)	0.0	(0.4)	1.13
	Spoken	0.3 (0.4)	0.0	(0.4)	1.12
	Letter	0.4 (0.9)	0.0	(0.4)	2.30
Reaction times (ms)					
Children	Tone	820 (127)	819	(199)	0.23
	Spoken	740 (121)	737	(174)	0.68
	Letter	568 (90)	552	(117)	0.10
Adults	Tone	660 (72)	650	(86)	0.60
	Spoken	637 (108)	605	(129)	1.13
	Letter	415 (65)	402	(40)	2.5

**Table 3:** Summary of *r* and *p* values (two-tailed) for correlations between the three continuous performance tasks (CPTs) and age, nonverbal IQ (NVIQ), and digit span forward and backward (raw scores). Where *p* (uncorrected) is ≤ .05, *r* has been bolded.

	Age		NVIQ		Digit span (forward)		Digit span (backward)	
CPT	*r*	*p*	*r*	*p*	*r*	*p*	*r*	*p*
Tone	**−.68**	**< .001**	−.02	.93	**−.49**	**.02**	−.31	.18
Spoken	**−.45**	**.05**	.03	.92	−.29	.22	−.19	.42
Visual	−.30	.21	.18	.45	.15	.54	−.12	.61
